# Dormancy dynamics and dispersal contribute to soil microbiome resilience

**DOI:** 10.1098/rstb.2019.0255

**Published:** 2020-03-23

**Authors:** Jackson W. Sorensen, Ashley Shade

**Affiliations:** 1 Department of Microbiology and Molecular Genetics, Michigan State University, East Lansing, MI 48824, USA; 2 Department of Plant, Soil and Microbial Sciences, Michigan State University, East Lansing, MI 48824, USA; 3 Program in Ecology, Evolutionary Biology and Behavior, Michigan State University, East Lansing, MI, USA

**Keywords:** disturbance ecology, resuscitation, microbial ecology, community assembly, resistance, immigration

## Abstract

In disturbance ecology, stability is composed of resistance to change and resilience towards recovery after the disturbance subsides. Two key microbial mechanisms that can support microbiome stability include dormancy and dispersal. Specifically, microbial populations that are sensitive to disturbance can be re-seeded by local dormant pools of viable and reactivated cells, or by immigrants dispersed from regional metacommunities. However, it is difficult to quantify the contributions of these mechanisms to stability without, first, distinguishing the active from inactive membership, and, second, distinguishing the populations recovered by local resuscitation from those recovered by dispersed immigrants. Here, we investigate the contributions of dormancy dynamics (activation and inactivation), and dispersal to soil microbial community resistance and resilience. We designed a replicated, 45-week time-series experiment to quantify the responses of the active soil microbial community to a thermal press disturbance, including unwarmed control mesocosms, disturbed mesocosms without dispersal, and disturbed mesocosms with dispersal after the release of the stressor. Communities changed in structure within one week of warming. Though the disturbed mesocosms did not fully recover within 29 weeks, resuscitation of thermotolerant taxa was key for community transition during the press, and both resuscitation of opportunistic taxa and immigration contributed to community resilience. Also, mesocosms with dispersal were more resilient than mesocosms without. This work advances the mechanistic understanding of how microbiomes respond to disturbances in their environment.

This article is part of the theme issue ‘Conceptual challenges in microbial community ecology’.

## 1. Introduction

Ongoing changes to Earth's climate are projected to alter disturbance regimes and to pervasively expose ecosystems to stressors like elevated atmospheric greenhouse gases and increased temperatures [1]. Microbial communities, or *microbiomes*, provide vital ecosystem functions and are key players in determining ecosystem responses to environmental changes [2,3]. Understanding the mechanisms that underpin microbiome responses to environmental disturbances will support efforts to predict, and, potentially, manage, microbiomes for stable functions within their ecosystems.

In disturbance ecology, stability refers to consistent properties in the face of a stressor [4]. Here, we apply terms from disturbance ecology as they have been adopted in microbial ecology [5–7]. Stability includes components of both resistance and resilience. Resistance is the capacity of a system to withstand change in the face of a stressor, and its inverse is sensitivity. Resilience is the extent to which a system recovers following a disturbance, and is often expressed as a rate of change over time. Secondary succession is the process of community reassembly after a disturbance, and it can lead to either a state of recovery or an alternative stable state. Recovery is when a system fully returns to either its pre-disturbance state or is indistinguishable from a comparative control, and this term can be applied both to the state of the stressor and to the responsive community. Similarly, an alternative stable state is when the system does not return but rather assumes a different state. Together, resistance and resilience are the major quantifiable components of stability, and they can be calculated from community measurements of alpha diversity, beta diversity or function [6,8].

There are two related microbial mechanisms that support population persistence in the face of disturbance, and therefore contribute to community resistance, resilience and recovery. One mechanism is microbial dispersal, as successful immigrants can support resilience and recovery of sensitive populations. Across an interconnected landscape, microbial metacommunities are linked via dispersal, and so immigrants originate from the regional species pool [9–12]. A second important but less-considered mechanism is microbial dormancy dynamics [13,14]. Dormancy dynamics include initiation and resuscitation. Initiation into dormancy can support local survival of populations sensitive to the disturbance, and therefore support community resistance by stabilizing community structure. Resuscitation from dormancy can support resilience and recovery by re-seeding sensitive populations from the local dormant pool. Thus, while both dispersal and resuscitation can support microbiome stability, dispersed immigrants originate regionally while resuscitated members originate locally. After a disturbance, if sensitive populations are not repopulated via immigration or resuscitation, they will become locally extinct and contribute to necromass (aka relic DNA, [15]).

We designed a replicated time-series experiment to quantify the contributions of dormancy dynamics and dispersal to the response of a soil microbiome to a thermal press disturbance. We targeted a soil microbiome because terrestrial microbiomes are front-line responders to climate change and sequesters of carbon [2,3], and therefore an important constituent to understand for predicting ecosystem outcomes to environmental change. Also, soils harbour the highest known microbial diversity [16–18] and present a maximum challenge in deciphering microbiome responses to disturbance. Furthermore, a majority of the microbial cells or richness in soil is dormant [13,19], reportedly as high as 80%, representing a considerable pool of microbial functional potential. Finally, across heterogeneous soils, an average of 40% of the microbiome DNA was necromass that existed extracellularly [15]. This suggests that DNA-based methods of determining microbiome dynamics include both inactive and necromass reservoirs, and that there is need for increased precision to move forward to quantify mechanisms underpinning microbiome stability.

The mesocosm experiment reported here follows prior fieldwork in Centralia, PA [20–24]. Centralia is the site of an underground coal seam fire that ignited in 1962 and advances 5–7 m yr^−1^ along the coal seams [25,26]. The coal seams are highly variable in depth, but average 70 m below the surface [25], so as the fire advances underground it warms the overlying surface soils from ambient to mesothermal to thermal conditions. After the fire advances, previously warmed soils cool to ambient temperatures. In the field, we observed that previously warmed soils recovered towards reference soils in bacterial and archaeal community structure, with the exception of a slightly increased selection for Acidobacteria in the recovered soils (attributable to lower soil pH after coal combustion, [20]). However, during fire impact, there was high divergence among soil communities, and we hypothesized that differences in dormancy dynamics (e.g. different members resuscitating and initiating priority effects during the stress) may explain the divergences. We also hypothesized that resuscitation would shift community structure during the thermal disturbance, but that resuscitation and dispersal would together support resilience after the disturbance subsided. Therefore, in this experiment, we aimed to control dispersal, and also to quantify activity dynamics and determine their consistency and test our hypotheses.

## 2. Material and methods

### (a) Soil collection, mesocosm design and soil sampling

Eight kilograms of soil was collected in Whirlpack bags from the top 10 centimetres of a reference site in Centralia, PA (site C08, 40 48.084 N, 076 20.765 W) on 31 March, 2018. The site is temperate with the following chemical–physical properties: organic matter 4.8%; nitrate 7.9 ppm; ammonium 20.5 ppm; pH 5; sulfur 19 ppm; potassium 69 ppm; calcium 490 ppm, magnesium 59 ppm; iron 110 ppm, and phosphorus 395 ppm. The ambient soil temperature when collected was 4°C. The sample was stored at 4°C until the experiment was initiated. Soil was sieved through a 4 mm mesh, homogenized, and approximately 300 g were dispensed into 15 autoclaved quart-sized glass canning jars that were used as mesocosms (Ball). The homogenized soil sample intentionally was used in all 15 mesocosms to assess the reproducibility of community temporal dynamics starting from the same soil source. Per cent soil moisture was determined by massing and drying. Each mesocosm was massed weekly to assess evaporation and any loss of water mass was replaced with sterile water to maintain per cent soil moisture throughout the experiment. Sterile metal canning lids were secured loosely to prevent anaerobiosis. All set-up and manipulation of the mesocosms was performed in a Biosafety Level 2 cabinet (ThermoScientific 1300 Series A2) and we used aseptic techniques.

**Figure 1.**
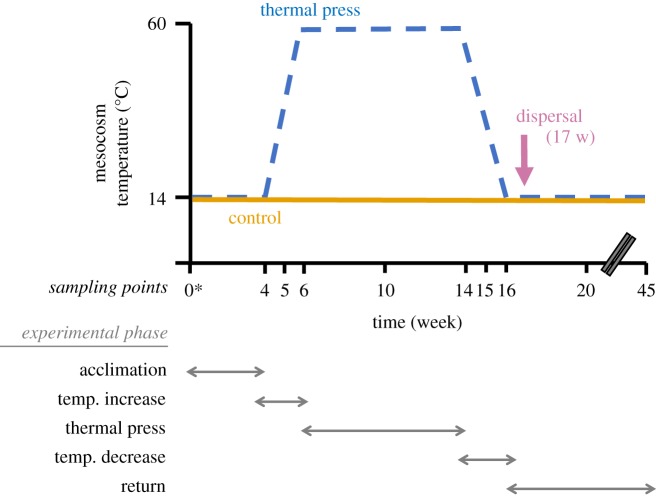
Experimental design of the study. At time 0 (indicated by the asterisk), reference temperate soil (0–20 cm depth from surface) was homogenized and divided among fifteen 1 l glass mesocosms that were maintained at ambient moisture through the experiment. Nondestructive sampling of each mesocosm proceeded from week 4 onwards as indicated by the *x*-axis. Unwarmed Control mesocosms (solid gold line, *n* = 6) were maintained at 14°C, which was ambient soil temperature at the time of collection. Disturbed mesocosms (dashed blue line, *n* = 9, including Disturbance and Disturbance + Immigration groups) were acclimated for four weeks at 14°C, increased to 60°C over two weeks, maintained at 60°C as a thermal press disturbance for eight weeks, then decreased back to 14°C over two weeks, and finally maintained for a total of 45 weeks. Four of the disturbance mesocosms received homogenized soil slurry from Control mesocosms as a dispersal event at week 17, after the thermal press was released (Disturbance + Immigration treatment; see methods). Note the break in the *x*-axis time scale between weeks 20 and 45.

Mesocosms first were acclimated at 14°C to mimic the ambient soil temperature at the typical time of fall soil collection and to coordinate with our previous field study [20]. Acclimation proceeded for four weeks in a cooling incubator (Fischer Scientific Isotemp), and then soils were divided into three treatment groups (figure 1). Six unwarmed control mesocosms (‘Control’) were maintained at 14°C for the duration of the experiment. Nine warmed mesocosms (‘Disturbance’) were subjected to a 12 week disturbance regime to simulate a press thermal disturbance. First, the temperature was gradually increased to 60°C, by 3°C to 3.5°C daily increments over two weeks. Second, the temperature was maintained at 60°C for eight weeks. Sixty degrees was chosen because it was close to the observed maximum thermal temperature that we have measured in surface soils impacted by the Centralia coal seam fire [20]. Next, the temperature was gradually decreased to 14°C, by 3°C to 3.5°C daily increments over two weeks. Finally, the mesocosms were maintained at 14°C for four weeks until the penultimate sampling. From the nine disturbed mesocosms, four were randomly selected for the dispersal treatment (‘Disturbance + Immigration’). These four disturbed mesocosms received a dispersal event one week after the temperature was recovered to 14°C after the thermal disturbance. Each was inoculated with 0.5 ml of a 10% weight by volume soil slurry made from a composite soil sample from the six unwarmed control mesocosms, and then gently mixed with a sterile spatula. Using quantitative polymerase chain reaction (qPCR) data from control mesocosms at week 16, we estimate that approximately 6.37 × 10^6^ cells were dispersed into each Disturbance + Immigration mesocosm. We used soil from the control mesocosms to simulate dispersal from similar, adjacent soils to repopulate disturbed communities, as expected in the field. Finally, all mesocosms were left undisturbed at 14°C for another 25 weeks prior to the final 45-week sampling. During the final 25 week incubation, per cent moisture was not monitored.

Mesocosms were non-destructively sampled after 4, 5, 6, 10, 14, 15, 16, 20 and 45 weeks of incubation. At each time point, approximately 15 g soil was removed from a mesocosm, of which approximately 13 g was flash-frozen in liquid nitrogen for RNA preservation and stored at −80°C until RNA/DNA co-extraction.

### (b) RNA/DNA co-extraction

To obtain RNA and DNA from the same cell pool, we minimally modified a manual coextraction protocol originally published by [27]. For each sample, 0.5 g of flash-frozen soil was added to Qiagen PowerBead Tubes containing 0.70 mm garnet beads. Next, 500 µl of a 5% cetyl trimethylammonium bromide/phosphate buffer and 500 µl of phenol : chloroform : isoamyl alcohol were added to each PowerBead tube. Cells were then lysed using a Model 607 MiniBeadBeater-16 (BioSpec Products Inc.) for 30 s, followed by a 10 min centrifugation at 10 000*g* and 4°C. The top aqueous layer was transferred to a fresh tube and 500 µl chloroform : isoamyl alcohol was added. The tubes were inverted several times to form an emulsion before a 5 min centrifugation at 16 000*g* and 4°C. The top aqueous layer was transferred to a clean 1.5 ml centrifuge tube. Nucleic acids were precipitated by adding two volumes of a 30% PEG6000 1.6 M NaCL solution, inverting several times to mix, and incubating on ice for 2 h. After incubation, nucleic acids were pelleted by a 20 min centrifugation at 16 000 *g* and 4°C. The supernatant was removed from each tube and one ml of ice-cold ethanol was added to the pelleted nucleic acids. Tubes were centrifuged for 15 min at 16 000*g* and 4°C, and the ethanol supernatant was removed. Pelleted nucleic acids were left to air dry before resuspending in 30 µl of sterile DEPC-treated water.

To purify the RNA, co-extracted nucleic acids were diluted 1 : 100 before treatment with Ambion Turbo DNA-free DNase kit, using the robust treatment option in the manufacturer's instructions. Extracted nucleic acids were mixed with 0.1 volumes of the 10X Turbo DNase Buffer and 3 µl of TURBO Dnase enzyme (six units total) and incubated at 37°C for 30 min. After incubation, 0.2 volumes of DNase inactivation reagent was added and incubated for 5 min at room temperature before a 5 min centrifugation at 2000*g* and room temperature. The treated supernatant was removed and used as the template for reverse transcription (RT). RNA purity was assessed by PCR (see below for details) and showed no amplification. RT was performed with random hexamers using the SuperScript III First-Strand Synthesis System for RT-PCR(Invitrogen) per manufacturer's instructions.

PCR of cDNA and no-RT controls was performed using the Earth Microbiome Project 16S rRNA gene V4 primers(515F 5′-GTGCCAGCMGCCGCGGTAA-3′, 806R 5′-GGACTACHVGGGTWTCTAAT-3′) [16,28]. Temperature cycling was as follows: 94°C for 4 min followed by 30 cycles of 94°C for 45 s, 50°C for 60 s and 72°C for 90 s followed by a final elongation step at 72°C for 10 min. Products were visualized using gel electrophoresis.

### (c) 16S rRNA and 16S rRNA gene sequencing and processing

Here, for simplicity we use ‘microbiome’ to refer to the bacterial and archaeal community members captured by amplifying and Illumina sequencing of the 16S ribosomal RNA and DNA (rRNA gene). Library preparation and sequencing was performed by the Michigan State University Genomics Core Research Facility. A single library was prepped using the method in Kozich *et al.* [29]. PCR products were normalized using Invitrogen SequalPrep DNA Normalization Plates. This library was loaded onto 4 separate Illumina MiSeq V2 Standard flow cells and sequenced using 250 bp paired end format with a MiSeq V2 500 cycle reagent cartridge. Base calling was performed by the Illumina Real Time Analysis (RTA) V1.18.54.

All samples were first checked for any contaminating primer sequences using cutadapt [30], before being processed together using the USEARCH pipeline [31,32]. Briefly, paired end reads were merged using -fastq_mergepairs and then dereplicated using -fastx_uniques. Reads were clustered *de novo* at 97% identity and then the original merged reads were mapped to the representative sequences of each cluster. Each operational taxonomic unit (OTU) was classified using SINTAX [33] and with the Silva database (v. 123, [34]).

### (d) Designating total and active communities

Each RNA and DNA sample was rarefied to 50 000 reads in R using the vegan package v. 2.5–4 [35] discarding any samples which did not contain sufficient reads (electronic supplementary material, figure S1). Samples for which either the RNA or DNA did not have 50 000 reads were omitted from the analysis presented here (12 out of 135 in total). The Total community was defined as the community recovered in the DNA reads. The Active community was defined per sample, using the DNA read numbers of those taxa that had a 16S rRNA : rRNA gene ratio greater than 1 in each sample [36]. Consequently, while every sample was initially rarefied to 50 000 reads, each sample's Active community varied slightly in total reads. Finally, we did not include taxa that had undefined rRNA : rRNA gene ratios (‘phantoms’) in the analysis (electronic supplementary material, figure S2, see discussion in the electronic supplementary material).

### (e) Quantitative polymerase chain reaction

qPCR was performed on the V4 region of the 16S rRNA gene and conducted in a BioRad CFX qPCR machine using the Absolute QPCR Mix, SYBR Green, no ROX (Thermo Scientific). Each reaction contained 12.5 µl of the 2X Absolute QPCR Mix, 1.25 µl each of 10 µM primers 515F and 806R, 3 µl of template DNA and 2 µl of PCR grade water. Temperature cycling conditions were as follows: 15 min at 95°C, followed by 39 cycles of 94°C for 45 s, 50°C for 60 s and 72°C for 90 s, followed by a final elongation step at 72°C for 10 min. Fluorescence was measured in each well at the end of every cycle. Extracted gDNA from *E. coli* MG1655 was used for the standard curve, and was run in triplicate with every plate. Samples were run in duplicate across different plates and those that amplified after the lowest point of the standard curve (27 copies per reaction) were treated as zeroes. No template controls were included in every qPCR plate and they never amplified. Amplification specificity was assessed by melt curve (60°C to 95°C, 0.5°C increments).

### (f) Calculating resistance and resilience of community structure

We calculated resistance and resilience as described in Shade & Peter [6] and Orwin & Wardle [8]. These are unitless metrics that have a theoretical range from −1 to 1. Resistance of the Active community structure at week 10 was calculated for every disturbed mesocosm using equation (2.1):

2.1 $$\text{RS}=1-\;\frac{2\;*\;|y_{c}-y_{d}|}{y_{c}+|y_{c}-y_{d}|},$$

where *y_c_* is the mean Bray–Curtis similarity for Control mesocosms at week 10 compared to week 4 (pre-disturbance), and *y_d_* is the individually calculated Bray–Curtis similarity of each disturbed mesocosm at week 10 to week 4. Resilience of the Active community in each disturbed mesocosm was calculated for the observed secondary succession (weeks 16–45) as well as the initial (weeks 16–20) and the long-term (weeks 20–45) secondary succession using equation (2.2):

2.2 $$\text{RL}=\frac{2\;*\;|y_{c,s}-y_{d,s}|}{\left(\left|y_{c,s}-y_{d,s}|+|y_{c,e}-y_{d,e}\right|\right)}-1,$$

where *s* is the start of the secondary succession and *e* is the end, *y_c_*_,*s*_ is the mean Bray–Curtis similarity of Control mesocosms at week *S* to week 4 (pre-disturbance), *y_d_*_,*s*_ is the Bray–Curtis similarity of each disturbed mesocosm at week *S* to week 4 (pre-disturbance), *y_c_*_,*e*_ is the mean Bray–Curtis similarity of Control mesocosms at week *e* to week 4, and *y_d_*_,*e*_ is the Bray–Curtis similarity of each disturbed mesocosms at week *e* to week 4.

### (g) Ecological statistics

Ecological analyses were performed in R [37]. The adonis and anosim function in the vegan package was used to perform PERMANOVAs [38] and ANOSIM respectively, to assess disturbance and immigration effects on community composition, and the betadisper function was used to quantify beta dispersion [39] with Tukey's honestly significant difference *post hoc* test across Control, Disturbance and Disturbance + Immigration treatments. Pairwise tests for alpha diversity (richness and Pielou's evenness), community size (i.e. 16S rRNA gene copies per gram of soil) and resilience values were performed using the Kruskal–Wallis test, with Dunn's *post hoc* correction for multiple comparisons when needed to assess differences between control, disturbance and immigration treatments. Principal coordinates analysis was used for ordination of pairwise sample differences based on Bray–Curtis dissimilarity. Procrustes superimposition (PROTEST) was performed using the procrustes function in the vegan package to compare community structure trajectories in direction and extent of change and a false discovery rate adjustment was used for multiple tests. Data visualizations were performed using ggplot2 [40]. Heatmaps were made using the heatmap.2 function in the gplots package [41].

To understand potential roles of dormancy initiation and resuscitation in driving community resistance and resilience, we distinguished between taxa that changed in their activity from taxa that changed in their detection over the course of the disturbance. Taxa that fell below detection (there was no rRNA gene detected in a particular sample) were coded differently for the heatmap than taxa that became inactive (rRNA : rRNA gene shifted from greater than 1 to less than 1). For the heatmap, we used the Active community for the input data, but coded taxa that fell below detection in the Total community as NAs to distinguish them from inactive taxa, which were coded as 0. Notably, taxa that fell below detection in the Total community could have been either active, inactive, or locally extinct. To conservatively attribute activity dynamics, we restricted the heatmap visualization only to the taxa that were among the 50 most abundant in Active samples over the course of the experiment.

Responsive taxa were those that changed in activity over secondary succession (between weeks 16, 20 and 45) by their 16S rRNA : rRNA gene ratio, either from less than 1 to greater than 1 or greater than 1 to less than 1. Immigrant taxa were undetected in all disturbed mesocosms at week 16, but detected in Control mesocosms at week 16 and Disturbance + Immigration mesocosms at either week 20 or week 45 while remaining undetected in the Disturbance mesocosms. Contributions of responsive and immigrant taxa to beta diversity were calculated as the Bray–Curtis dissimilarity attributed to the responsive taxa subset and divided by the total Bray–Curtis dissimilarity, both calculated from the Total (DNA) community, as done previously to assess the contributions of conditionally rare taxa [42] and the contributions of core taxa [43] to beta diversity. Briefly, to calculate the proportional contribution of any subset of taxa to observed Bray–Curtis similarity, the Bray–Curtis dissimilarity attributable to the subset of taxa is divided by the total Bray–Curtis dissimilarity calculated from the entire community. Because Bray–Curtis dissimilarity is the sum of the difference in abundances of taxa in two communities divided by the total abundance of the taxa in those two communities, one can calculate the contribution of a subset of taxa to the Bray–Curtis dissimilarity by only including the subset in the numerator while including the total community in the denominator. This approach is transferable to other resemblance metrics and not restricted to use with Bray–Curtis. The detailed code for this calculation is available on GitHub.

### (h) Data availability and code

Sequence workflows, OTU tables and statistical workflows to reproduce the analyses described here are available on GitHub (https://github.com/ShadeLab/PAPER_Sorensen_PhilTransB_2020). All raw sequence data are deposited in the NCBI Short Read Archive under BioProject PRJNA559185.

## 3. Results

### (a) Sequencing summary

In total, we sequenced 135 pairs of samples (cDNA and DNA) across nine timepoints and 15 mesocosms. We rarefied all samples to 50 000 reads, and removed those samples with fewer than 50 000 reads. This resulted in the removal of 12 samples and left 53 unwarmed Control, 36 Disturbance and 34 Disturbance + Immigration pairs of samples. After rarefaction, sample richness ranged from 84 to 4108, with 16 854 total OTUs observed, inclusive of both DNA and RNA datasets.

### (b) Overarching responses to the thermal press disturbance

**Figure 2.**
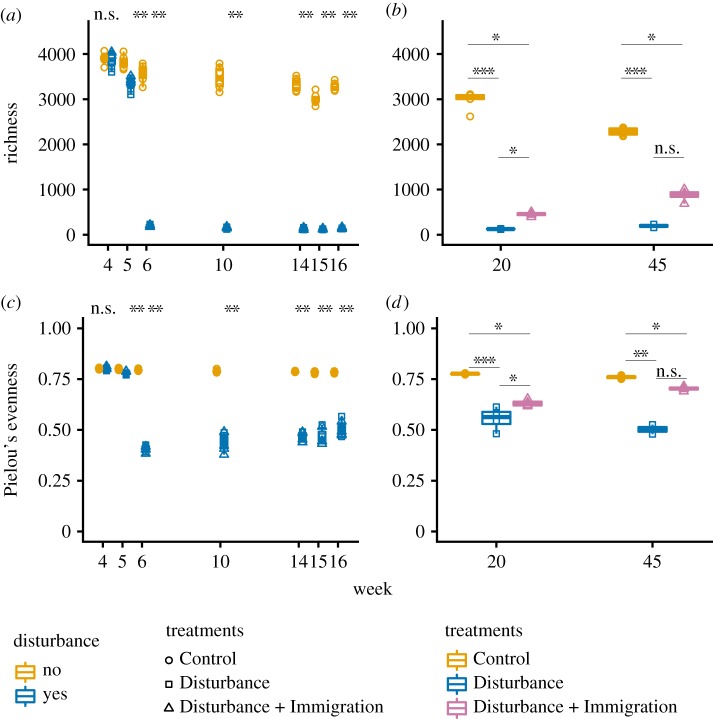
Changes in alpha diversity over the disturbance experiment. Alpha diversity was assessed using operational taxonomic units (OTUs) clustered at 97% sequence identity, after 16S rRNA gene sequencing and rarefaction to 50 000 sequences per sample. (*a*) Changes in the observed no. OTUs (richness) in Control (gold, circles) and Disturbance (blue, squares and triangles) mesocosms over the thermal press (weeks 4–16). (*b*) Changes in richness in Control (gold circles), Disturbance (blue squares) and Disturbance + Immigration (pink triangles) mesocosms over the recovery period, weeks 20–45. The Disturbance + Immigration mesocosms received a dispersal event at week 17. (*c*) Changes in evenness over weeks 4–16. (*d*) Changes in evenness over weeks 20–45. Asterisks indicate significant differences by a Kruskal–Wallis test (n.s., not significant; *p < 0.1, **p < 0.01, ***p < 0.001, with a Dunn correction for multiple comparisons in *b* and *d*).

**Figure 3.**
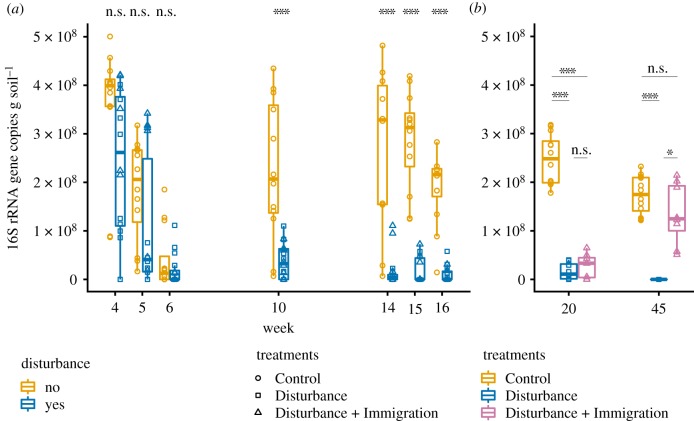
Changes in community size over the disturbance experiment. Community size was estimated using qPCR of the 16S rRNA gene and standardized per g soil from which nucleic acids were extracted. (*a*) Changes in the 16S rRNA gene copies in Control (gold, circles) and disturbed (blue, squares and triangles) mesocosms over the thermal press (weeks 4–16). (*b*) Changes in the 16S rRNA gene copies in Control, Disturbance (blue squares) and Disturbance + Immigration (pink triangles) mesocosms over the recovery period, weeks 20–45. The Disturbance + Immigration mesocosms received a dispersal event at week 17. Asterisks indicate significant differences by a Kruskal–Wallis test (n.s., not significant; *p < 0.1, ***p < 0.001, with a Dunn correction for multiple comparisons in *b*).

Total community richness responded consistently and as expected to the thermal press disturbance. There was a notable bottle effect of maintaining field soil in mesocosms, indicated by the gradual decrease in richness over time in the unwarmed Control treatment (figure 2*a*,*b*). In the Disturbance treatment, there was a modest but statistically supported decrease in richness one week after warming from 14°C to 37°C (week 5 all Disturbance v. Control comparison, Kruskal–Wallis test, *p* = 0.003), and then a more substantial decrease after warming to 60°C at week 6 (Kruskal–Wallis test, *p* = 0.002). Disturbance community size decreased over weeks four to seven and then maintained at a median of 1.03 × 10^7^ rRNA gene copies g soil^−1^ (figure 3). Control communities decreased until week 7 (bottle effect) and then increased rapidly by week 10 and generally stabilized at median of 2.98 × 10^8^ 16S rRNA gene copies g soil^−1^ (figure 3*a*). Together, these results show that the warming treatment acted as an environmental filter, resulting either in death or population decreases past the limits of detection for taxa that were otherwise fit in unwarmed conditions. Furthermore, there was a weak increase in richness after the dispersal event in the Disturbance + Immigration treatment, relative to the Disturbance treatment (Kruskal–Wallis test *p* = 0.088 at week 20, and *p* = 0.168 at week 45), and this increase was also observed for community size, which approaches that of the unwarmed control (Kruskal–Wallis test Control versus Disturbance + Immigration *p* = 0.11, Control versus Disturbance *p* = 0.0004, Disturbance versus Disturbance + Immigration *p* = 0.013) (figure 3*b*). This suggests that the dispersal treatment was effective in promoting the process of recovery in richness and community size. Importantly, Disturbance and Disturbance + Immigration mesocosms were not significantly different in either richness nor community size prior to the immigration event (electronic supplementary material, tables S1 and S2) However, disturbed mesocosms did not completely recover richness to the level of the ambient Controls, even by week 45 (figure 2*b*). Evenness followed the same overarching patterns as richness (figure 2*c*,*d*).

**Figure 4.**
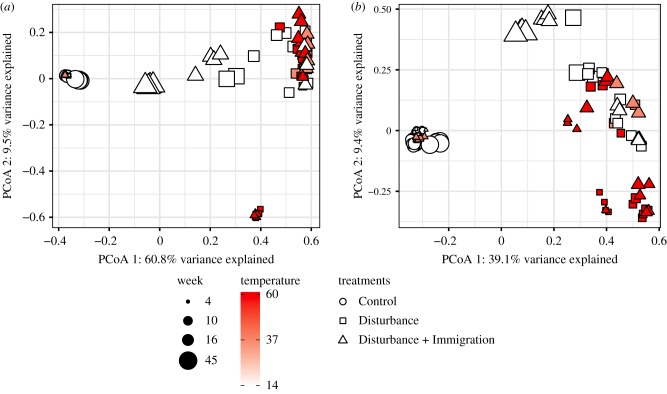
Changes in beta diversity over the disturbance experiment. Pairwise differences in community structure was quantified using pairwise Bray–Curtis dissimilarity and then ordinated using principal coordinates analysis (PCoA). Time is shown by symbol size, and mesocosm temperature is indicated by heat colours, with the brightest red indicating the warmest time point. Control mesocosms are circles, Disturbance are squares and Disturbance + Immigration are triangles. (*a*) PCoA of the Total community, assessed using sequencing of the 16S rRNA gene. (*b*) PCoA of the Active community, including only OTUs that had 16S rRNA : rRNA gene > 1.

**Figure 5.**
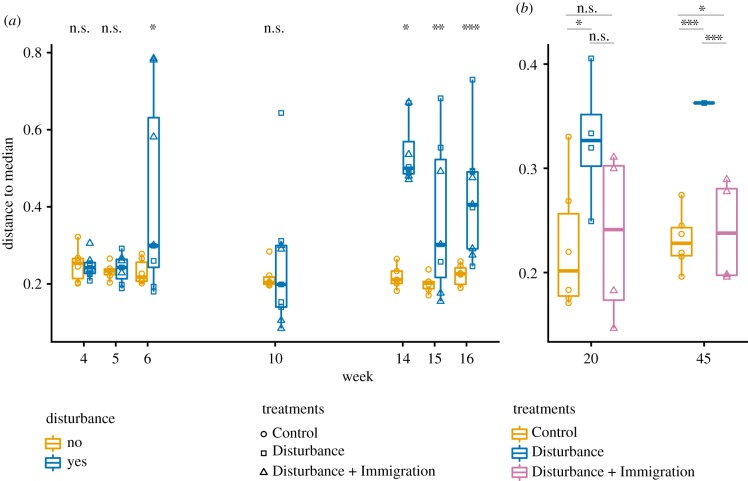
Changes in beta dispersion over the disturbance experiment. Beta dispersion, an indicator of variability in community structure, was quantified using the distance to the median in ordination space (figure 4), which was constructed based on Bray–Curtis dissimilarity. (*a*) Changes in beta dispersion in Control (gold, circles) and Disturbance (blue, squares and triangles) mesocosms over the thermal press (weeks 4–16). (*b*) Changes in beta dispersion in Control, Disturbance (blue squares) and Disturbance + Immigration (pink triangles) mesocosms over the recovery period, weeks 20–45. The Disturbance + Immigration mesocosms received a dispersal event at week 17. Asterisks indicate significant differences with a Tukey's honestly significant difference *post hoc* test (n.s., not significant; *p < 0.1, **p < 0.01, ***p < 0.001). Note differences in *y*-axis ranges between (*a*) and (*b*).

We compared community structure across treatments for the Total community dataset, (rRNA gene; 14 159 OTUs) and the Active dataset (rRNA:rRNA gene > 1; 6693 = OTUs). There were clear and consistent shifts in beta diversity in the disturbed mesocosms (*n* = 9, inclusive of Disturbance and Disturbance + Immigration), as well as high reproducibility among replicates in community structure within treatments as shown by the overlap of symbols per treatment and timepoint in the ordination (figure 4). As compared to the Controls, the disturbed mesocosms had increased betadispersion (variability in community structure) starting at week 6 onwards, with the exception of week 10 (figure 5). Over the experiment, disturbed mesocosms had distinct community structures compared to Control (disturbed v. Control PERMANOVA PsuedoF = 63.87, Rsqr = 0.345, *p* = 0.001 for Total communities, and PsuedoF = 35.97, Rsqr = 0.229, *p* = 0.001 for Active communities, all timepoints). Control communities were relatively stable over the study, while disturbed communities changed directionally, and were significantly different from Control communities after a single week of warming (week 5 Control versus disturbed PERMANOVA PsuedoF = 3.06, Rsqr = 0.218, *p* = 0.001 for Total community and PsuedoF = 2.88, Rsqr = 0.208, *p* = 0.001 for Active community, Week 4 PERMANOVA *p* > 0.05, electronic supplementary material, table S3). Disturbed communities continued to shift with temperature during the course of the experiment, and then shifted slightly back towards the Control after the stressor was released and Disturbance and Disturbance + Immigration communities had similar structures during the press (electronic supplementary material, table S4). Though no disturbed mesocosms fully recovered to overlap with the Control communities, the Disturbance + Immigration mesocosms were more similar to the Control than the Disturbance mesocosms without dispersal (figures 2*b*, 3*b* and 4). Across all treatments, Total communities and Active communities were synchronous in their temporal trajectories (Mantel *R* = 0.943, *p* = 0.001 on 999 permutations; Protest Sum of Squares = 0.238, *R* = 0.873, *p* = 0.001), but there was higher betadispersion in the disturbed treatments for the Active communities (comparing Total v. Active for disturbed mesocosms, Kruskal–Wallis *p* = 0.029). This suggests that there was Active community variability masked by the contributions of dead and dormant taxa to the Total community.

Replicate disturbed mesocosms (again, inclusive of Disturbance and Disturbance + Immigration) had highly reproducible responses during the press. They had high overlap in membership and overall synchronous trajectories (i.e. changes in community structure through time), even after the immigration event at week 16 (33 of 36 PROTEST all *R* > 0.89 and false-discovery rate adjusted *p*-values < 0.05).

### (c) Resistance and resilience

**Figure 6.**
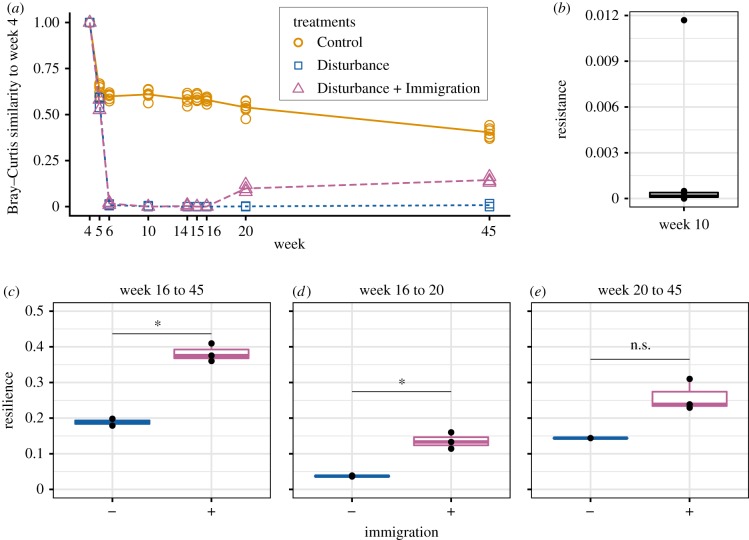
Resistance and resilience of soil mesocosm communities to a thermal press. (*a*) Temporal series of community divergence from pre-disturbance community (week 4) in Control (gold solid line), Disturbance (blue short dashed line) and Disturbance + Immigration (pink long dashed line) to calculate resistance and resilience. (*b*) Resistance of disturbed mesocosms at week 10, the time point of maximum community change after the thermal press begins. (*c*–*e*) Resilience of disturbed mesocosms without (−) and with (+) immigration, calculated after the thermal press is released (week 16) for the (*c*) full recovery to week 45, (*d*) initial recovery to week 20, and also for (*e*) long-term recovery from weeks 20 to 45. Asterisks indicate significant differences by a Kruskal–Wallis test (n.s., not significant; *p < 0.1).

For the Active community, we calculated resistance and resilience of the disturbed mesocosms relative to the Control using community divergence from the first sampling time (week 4, end of acclimatization period) as the reference (figure 6*a*). Even in the Control communities, there was an initial drop in similarity between weeks 4 and 5, which we attribute to incomplete acclimatization and a bottle effect. However, after that, the Control communities remain relatively stable with no additional divergence, while the disturbed communities decrease to their maximum divergence at week 10 (60°C).

Disturbance + Immigration communities converge slightly after the dispersal event. Overall resistance was low (figure 6*b*), and resilience reached its maximum, 0.41, in the immigration treatment between weeks 16 (the time point at which the thermal press was released) and the final week 45, but ranged from a minimum of 0.04 between weeks 16 and 20 in the Disturbance without immigration treatment (figure 6*c–e*). Immigration enhanced resilience from week 16 to week 20 (Kruskal–Wallis *p*-value 0.034) and from week 16 to week 45 (Kruskal–Wallis *p*-value 0.083), but not from week 20 to 45, possibly because of insufficient power (Kruskal–Wallis *p*-value 0.180). Notably, there were only two Disturbance mesocosm replicates (out of five) that met the rarefaction threshold for week 45.

We wanted to assess the relative contributions of taxa that activate or inactivate after the disturbance subsides to the overall beta diversity (weeks 16–45). We also wanted to assess the relative contributions of taxa that colonized after dispersal. We calculated the relative contribution of activity dynamics by identifying taxa that switched between an active and inactive state during secondary succession. We found that these dynamically active taxa contributed 11.7–58.9% (median 28.6%) of the observed beta diversity, while immigrants contributed 7.9–26.3% (median 14.7%) of the observed beta diversity during the same time period**.**

### (d) Activity dynamics of abundant taxa

**Figure 7.**
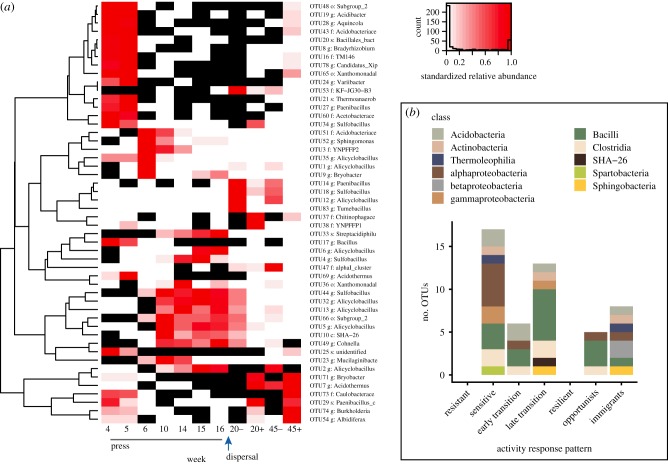
The activity dynamics of the 50 most abundant taxa in response to the press disturbance. (*a*) Heatmap and dendrogram of abundant taxa reveal common patterns of detection and activity. Black cells are taxa that were undetected (coded as NA) in the 16S rRNA gene (DNA) community, and white cells are taxa that were detected in the DNA but had 16S rRNA:rRNA gene < 1 (inactive, coded as 0). The heat gradient indicates each taxon's abundance relative to its maximum observed in disturbance treated mesocosms during the experiment. Immigration is indicated for weeks 20 and 45 by minus (no) and plus (yes) signs. (*b*) Summary of activity response patterns to the disturbance of the top 50 taxa, including resistant, sensitive, early and late transition, resilient, opportunist, and immigrant taxa. Definitions of each of these categories of taxa are found in the main text.

We investigated the activity dynamics of the top 50 most abundant taxa within the Active communities, and distinguished taxa that became inactive (rRNA : rRNA gene < 1, white cells in figure 7*a*) from taxa that fell below detection (rRNA gene = 0, black cells in figure 7*a*, see Methods for details). Within this set of 50, we detected no purely resistant taxa that were consistently active throughout the experiment. This finding agrees with the analyses showing low resistance (figure 6*b*) and substantial shifts in the disturbed communities (figure 5). We detected 17 taxa that were sensitive to the disturbance (figure 7*b*). Sensitive taxa were active prior to the warming but became inactive or dropped below detection during the warming, and then did not reactivate. We also detected 19 transition taxa that were inactive prior to the warming, active during the warming, and then became inactive after the stressor was released. Because there was no external dispersal into the system, these thermotolerant taxa were likely in the dormant pool of the soil. We could divide these responses generally into early and late transition taxa. There were six early transition taxa that became active during week 5 or 6 of the experiment, but then became inactive at weeks 10 and 14. There were also 13 late transition taxa that remained inactive during weeks 5 and 6 but became active during weeks 10 and 14.

Among the top 50 Active taxa, we did not detect purely resilient taxa that were active prior to the warming, became inactive during the warming, but then reactivated after the return to ambient temperature. This suggests that dormancy strategies responsive to warming were not a substantial contributor to member preservation, nor to eventual re-seeding. Instead, opportunists and immigrants facilitated resilience in the mesocosms. The opportunists were defined as inactive or below detection prior to and during the warming, but then activated after the temperature returned, probably owing to resuscitation, and there were five taxa in this category. Eight immigrants were generally active prior to the warming, dropped to below detection or became inactive during the warming, and then in the end, were active again only in the Disturbance + Immigration treatment (and not in the Disturbance mesocosms without Immigration).

## 4. Discussion

Our results show that both dispersal and local dormancy dynamics, including activation and inactivation, can contribute to overarching patterns of community resilience. The dispersal event simulated in this experiment posed an optimistic scenario: well-mixed, control soils were mixed into disturbed soils to maximize the volume of the disturbed soil that came into contact with the inoculum. Regardless, by all metrics (beta diversity, alpha diversity, community size), immigration was impactful. These data directly show that dispersal can augment resilience towards recovery, supporting our hypothesis. Given that the influences of dispersal on community assembly have been investigated previously (often indirectly for bacterial and archaeal microbiomes, as inferred from the contributions of stochastic or neutral processes, e.g. [20,44–47]), this result is in agreement with the consensus of the literature that dispersal and dispersal limitation can matter for assembly [48–50].

A new result is that local resuscitation also contributes to microbiome community transitions during disturbance, and to resilience after the stress is released. Among the most abundant taxa, there were near equal numbers of taxa that contributed to resilience via resuscitation and to resilience via immigration. While, the influence of resuscitation on resilience was not as impactful as that of dispersal (figure 6), changes in activity dynamics contributed 28.9% to the observed beta diversity during secondary succession. Therefore, both mechanisms—local resuscitation and regional immigration—contribute to microbiome stability, but potentially to different extents. The microbial dormant pool is important for maintaining microbial diversity [51] and has evolutionary implications for traits that persist within inactive populations [52]. To make more explicit the role of dormancy dynamics for community disturbance responses (e.g. [53]), the phenomenon of the ‘storage effect’ underpins modern coexistence theory [54] and refers to the ability of competing species to coexist when their growth and activities are separately partitioned over time, typically in dynamic environments [55]. Given the severity of the thermal stressor in Centralia and in this experiment, our results suggest that the soil microbial dormant pool is deep, in that it contains functionality for distinctive conditions, like thermal stress, that are not within the expected range of environmental variability. Our finding support other studies which have found thermophiles in unexpected environments, such are arctic sediments and temperate soils [56–58].

Alternatively, it could be that, rather than local resuscitation, extremely rare but active taxa that were below the limits of detection grew rapidly and repopulated to become among the most active and abundant taxa. These data cannot rule out this possibility, and, if true, it would suggest an interesting role for release of rare taxa from competition (via death or inactivation of the competitors sensitive to the warming) in driving post-disturbance assembly. However, given that no resistant taxa were detected that could withstand the wide temperature range in the experiment, conditional rarity may be a less common scenario than opportunistic resuscitation.

Another goal of the experiment was to understand the reproducibility of member resuscitation given the press disturbance, and from the same soil. Because we observed high divergence in the hot soil communities in Centralia that was not attributable to any measured environmental variable, including temperature [20], we hypothesized that stochastic resuscitation could initiate priority effects (e.g. [10]), leading to divergent hot communities. However, we did not see the strongest differences in beta dispersion between Control and disturbed mesocosms until the press was subsiding (weeks 15 and 16 in figure 5). This, along with the overall strongly-correlated trajectories of disturbed community structures, suggest that the disturbance responses were consistent across disturbed mesocosms and do not support our hypothesis that priority effects (initiated by different resuscitating membership) determines community structure during the press. Therefore, we interpret that resuscitation in response to the thermal stress was largely deterministic, and that observed divergences among hot soil communities in the field may be instead attributed to either difference in local edaphic factors that were unmeasured, different structures of the underlying dormant pools, or stochasticity in regional dispersal during secondary succession.

Moving forward, there are several insights gleaned from this experiment. For soil, measuring dispersal in the field is difficult, given the various means by which microorganisms may arrive to a locality, including wind, ground water and invertebrate vectors. Therefore, controlled experimentation is needed to quantify the contributions of dispersal to secondary succession. However, measuring activity dynamics and estimating the dormant pool of microbes in field samples, while imperfect, is possible [19,36,59,60]. Because our experiment suggests a role of resuscitation in determining the community that thrives during the disturbance, and also an influence of resuscitation for secondary succession towards recovery, we recommend collecting member activity data. More generally, routine characterization of the dormant pool of soil microbes, including its stability, diversity and functions, can provide insights into the roles of these inactive taxa for disturbance responses.

Microbiome stability encompasses a progression along a trajectory, including a pre-disturbance community with a variance around a mean structure or a routine seasonal dynamic, a transition to an ephemeral community structure during the disturbance, and finally, after the disturbance is released, secondary succession towards either recovery or an alternative stable state. Longitudinal series of microbiome structure inclusive of all stages of this trajectory can be informative. Characterizing the full disturbance trajectory will allow for quantification of the different and potentially changing mechanisms that support stability (e.g. resuscitation, conditional rarity, immigration), and will facilitate prediction given new stressors. In our experiment, one week of stress was sufficient to observe community sensitivity (by week 5, the control and the disturbance treatments were statistically different), but 29 weeks after the stress was released was not sufficient to observe complete recovery, though it seems that recovery is possible given the trajectory towards the controls. We expect that this timeframe of response may be typical for many soils [61] and it can be used to inform future studies. Notably, while the objective of this study was to assess responses to elevated temperature, we expect that nutrient limitation was an outcome of the closed system experiment because we did not supplement it with resources. We expect microbial responses to nutrient limitation occurred in both control and disturbed mesocosms, and that nutrient limitation compounded with thermal stress in the disturbed mesocosms. Therefore, nutrient limitation may have contributed to incomplete recovery trajectory.

To conclude, this experiment shows both dispersal and dormancy dynamics can contribute to soil microbiome resilience in response to a press stress. Specifically, resuscitation of thermotolerant members contributed to microbiome transition during press, and then immigration provided a substantial boost to recovery beyond what was achieved with resuscitated opportunists. Because activity responses to the disturbance were consistent, these results suggest that predictive insights into microbiome resilience can be advanced more generally. We expect that accounting for mechanisms of local resuscitation and regional dispersal together will advance quantitative understanding of environmental microbiome stability.

## Supplementary Material

Supporting results, figures and tables

## Data accessibility

All sequence workflows, OTU tables and statistical workflows to reproduce the analyses described here are available on GitHub (https://github.com/ShadeLab/PAPER_Sorensen_PhilTransB_2020). All raw sequence data are deposited in the NCBI Short Read Archive under BioProject PRJNA559185.

## References

[1] IPCC. 2014 Climate Change 2014 . (10.1017/CBO9781107415324)

[2] Cavicchioli R et al 2019 Scientists’ warning to humanity: microorganisms and climate change. Nat. Rev. Microbiol. 17, 569–586. (10.1038/s41579-019-0222-5) 31213707 PMC7136171

[3] Singh BK, Bardgett RD, Smith P, Reay DS 2010 Microorganisms and climate change: terrestrial feedbacks and mitigation options. Nat. Rev. Microbiol. 8, 779–790. (10.1038/nrmicro2439) 20948551

[4] Pimm SL 1984 The complexity and stability of ecosystems. Nature 307, 321–326. (10.1038/307321a0)

[5] Allison SD, Martiny JBH 2008 Resistance, resilience, and redundancy in microbial communities. Proc. Natl Acad. Sci. USA 105, 11 512–11 519. (10.1073/pnas.0801925105) 18695234 PMC2556421

[6] Shade A et al 2012 Fundamentals of microbial community resistance and resilience. Front. Microbiol. 3, 417 (10.3389/fmicb.2012.00417) 23267351 PMC3525951

[7] Kearns PJ, Shade A 2018 Trait-based patterns of microbial dynamics in dormancy potential and heterotrophic strategy: case studies of resource-based and post-press succession. ISME J. 12, 2575–2581. (10.1038/s41396-018-0194-x) 29959406 PMC6194022

[8] Orwin KH, Wardle DA 2004 New indices for quantifying the resistance and resilience of soil biota to exogenous disturbances. Soil Biol. Biochem. 36, 1907–1912. (10.1016/j.soilbio.2004.04.036)

[9] Leibold MA et al 2004 The metacommunity concept: a framework for multi-scale community ecology. Ecol. Lett. 7, 601–613. (10.1111/j.1461-0248.2004.00608.x)

[10] Fukami T 2015 Historical contingency in community assembly: integrating niches, species pools, and priority effects. Annu. Rev. Ecol. Evol. Syst. 46, 1–23. (10.1146/annurev-ecolsys-110411-160340)

[11] Langenheder S, Berga M, Östman Ö, Székely AJ 2012 Temporal variation of β-diversity and assembly mechanisms in a bacterial metacommunity. ISME J. 6, 1107–1114. (10.1038/ismej.2011.177) 22158394 PMC3358023

[12] Nemergut DR et al 2013 Patterns and processes of microbial community assembly. Microbiol. Mol. Biol. Rev. 77, 342–356. (10.1128/MMBR.00051-12) 24006468 PMC3811611

[13] Lennon JT, Jones SE 2011 Microbial seed banks: the ecological and evolutionary implications of dormancy. Nat. Rev. Microbiol. 9, 119–130. (10.1038/nrmicro2504) 21233850

[14] Hawkes CV, Keitt TH 2015 Resilience vs. historical contingency in microbial responses to environmental change. Ecol. Lett. 18, 612–625. (10.1111/ele.12451) 25950733

[15] Carini P, Marsden PJ, Leff JW, Morgan EE, Strickland MS, Fierer N 2016 Relic DNA is abundant in soil and obscures estimates of soil microbial diversity. Nat. Microbiol. 2, 16242 (10.1101/043372) 27991881

[16] Thompson LR et al 2017 A communal catalogue reveals Earth's multiscale microbial diversity. Nature 551, 457–463. (10.1038/nature24621) 29088705 PMC6192678

[17] Locey KJ, Lennon JT 2016 Scaling laws predict global microbial diversity. Proc. Natl Acad. Sci. USA 113, 5970–5975. (10.7287/peerj.preprints.1451v1) 27140646 PMC4889364

[18] Louca S, Mazel F, Doebeli M, Parfrey LW 2019 A census-based estimate of Earth's bacterial and archaeal diversity. PLoS Biol. 17, e3000106 (10.1371/journal.pbio.3000106) 30716065 PMC6361415

[19] Blagodatskaya E, Kuzyakov Y 2013 Active microorganisms in soil: critical review of estimation criteria and approaches. Soil Biol. Biochem. 67, 192–211. (10.1016/j.soilbio.2013.08.024)

[20] Lee S-H, Sorensen JW, Grady KL, Tobin TC, Shade A 2017 Divergent extremes but convergent recovery of bacterial and archaeal soil communities to an ongoing subterranean coal mine fire. ISME J. 11, 1447–1459. (10.1038/ismej.2017.1) 28282042 PMC5437352

[21] Sorensen JW, Dunivin TK, Tobin TC, Shade A. 2018 Ecological selection for small microbial genomes along a temperate-to-thermal soil gradient. Nat. Microbiol. 4, 55–61. 30397342 10.1038/s41564-018-0276-6

[22] Dunivin TK, Shade A 2018 Community structure explains antibiotic resistance gene dynamics over a temperature gradient in soil. FEMS Microbiol. Ecol. 94, fiy016 (10.1093/femsec/fiy016) PMC6018995 29401285

[23] Kearns PJ, Shade A 2018 Trait-based patterns of microbiome succession in dormancy and heterotrophic strategy: case studies of resource-based and post-press disturbance. ISME J. 12, 2575–2581. (10.1038/s41396-018-0194-x) 29959406 PMC6194022

[24] Tobin-Janzen T, Shade A, Marshall L, Torres K, Beblo C, Janzen C, Lenig J, Martinez A, Ressler D 2005 Nitrogen changes and domain bacteria ribotype diversity in soils overlying the Centralia, Pennsylvania underground coal mine fire. Soil Sci. 170, 191–201. (10.1097/00010694-200503000-00005)

[25] Nolter MA, Vice DH 2004 Looking back at the Centralia coal fire: a synopsis of its present status. Int. J. Coal Geol. 59, 99–106. (10.1016/j.coal.2003.12.008)

[26] Elick JM 2011 Mapping the coal fire at Centralia, PA using thermal infrared imagery. Int. J. Coal Geol. 87, 197–203. (10.1016/j.coal.2011.06.018)

[27] Griffiths R, Whiteley A, O'Donnell A 2000 Rapid method for coextraction of DNA and RNA from natural environments for analysis of ribosomal DNA- and rRNA-based microbial community composition. Appl. Environ. Microbiol. 66, 5488–5491. (10.1021/ja00751a011) 11097934 PMC92488

[28] Caporaso JG, Lauber CL, Walters WA, Berg-Lyons D, Lozupone CA, Turnbaugh PJ, Fierer N, Knight R 2011 Global patterns of 16S rRNA diversity at a depth of millions of sequences per sample. Proc. Natl Acad. Sci. USA 108, 4516 (10.1073/pnas.1000080107) 20534432 PMC3063599

[29] Kozich JJ, Westcott SL, Baxter NT, Highlander SK, Schloss PD 2013 Development of a dual-index sequencing strategy and curation pipeline for analyzing amplicon sequence data on the MiSeq Illumina sequencing platform. Appl. Environ. Microbiol. 79, 5112–5120. (10.1128/AEM.01043-13) 23793624 PMC3753973

[30] Martin M 2011 Cutadapt removes adapter sequences from high-throughput sequencing reads. EMBnet J. 17, 10–12. (10.14806/ej.17.1.200)

[31] Edgar RC 2010 Search and clustering orders of magnitude faster than BLAST. Bioinformatics 26, 2460–2461. (10.1093/bioinformatics/btq461) 20709691

[32] Edgar RC, Flyvbjerg H 2014 Error filtering, pair assembly and error correction for next-generation sequencing reads. Bioinformatics 31, 3476–3482. (10.1093/bioinformatics/btv401) 26139637

[33] Edgar RC 2016 SINTAX: a simple non-Bayesian taxonomy classifier for 16S and ITS sequences. *bioRxiv*, 074161 (10.1101/074161)

[34] Quast C, Pruesse E, Yilmaz P, Gerken J, Schweer T, Yarza P, Peplies J, Glöckner FO 2013 The SILVA ribosomal RNA gene database project: improved data processing and web-based tools. Nucleic Acids Res. 41, D590–596. (10.1093/nar/gks1219) 23193283 PMC3531112

[35] Oksanen J et al 2019 vegan: community ecology package. R package version 2.5-6. See https://CRAN.R-project.org/package=vegan.

[36] Bowsher AW, Kearns PJ, Shade A 2019 16S rRNA/rRNA gene ratios and cell activity staining reveal consistent patterns of microbial activity in plant-associated soil. mSystems 4, e00003-19 (10.1128/msystems.00003-19) 30944883 PMC6445865

[37] R Core Team. 2019 R: a language and environment for statistical computing Vienna, Austria: R Foundation for Statistical Computing See https://www.R-project.org/.

[38] Anderson MJ 2001 A new method for non-parametric multivariate analysis of variance. Austral Ecol. 26, 32–46.

[39] Anderson MJ 2005 Distance-based tests for homogeneity of multivariate dispersions. Biometrics 62, 245–253. (10.1111/j.1541-0420.2005.00440.x) 16542252

[40] Wickham H 2009 Ggplot2: elegant graphics for data analysis. New York, NY: Springer-Verlag See http://ggplot2.org.

[41] Warnes GR et al. 2016 gplots: various R programming tools for plotting data R package version 3.0.1.1. See https://CRAN.R-project.org/package=gplots.

[42] Shade A, Jones SE, Caporaso JG, Handelsman J, Knight R, Fierer N, Gilbert JA 2014 Conditionally rare taxa disproportionately contribute to temporal changes in microbial diversity. mBio 5, e01371-14 (10.1128/mBio.01371-14) 25028427 PMC4161262

[43] Grady KL, Sorensen JW, Stopnisek N, Guittar J, Shade A 2019 Assembly and seasonality of core phyllosphere microbiota on perennial biofuel crops. Nat. Commun. 10, 4135 (10.1038/s41467-019-11974-4) 31515535 PMC6742659

[44] Ferrenberg S et al 2013 Changes in assembly processes in soil bacterial communities following a wildfire disturbance. ISME J. 7, 1102–1111. (10.1038/Ismej.2013.11) 23407312 PMC3660671

[45] Dini-Andreote F, Stegen JC, van Elsas JD, Salles JF. 2015 Disentangling mechanisms that mediate the balance between stochastic and deterministic processes in microbial succession. Proc. Natl Acad. Sci. USA 112, E1326–E1332. (10.1073/pnas.1414261112) 25733885 PMC4371938

[46] Burns AR, Zac Stephens W, Stagaman K, Wong S, Rawls JF, Guillemin K, Bohannan BJ 2016 Contribution of neutral processes to the assembly of gut microbial communities in the zebrafish over host development. ISME J. 10, 655–664. (10.1038/ismej.2015.142) 26296066 PMC4817674

[47] Zhou J et al 2013 Stochastic assembly leads to alternative communities with distinct functions in a bioreactor microbial community. mBio 4, e00584-12. (10.1128/mBio.00584-12) 23462114 PMC3585448

[48] Evans S, Martiny JB, Allison SD 2017 Effects of dispersal and selection on stochastic assembly in microbial communities. ISME J. 11, 176–185. (10.1038/ismej.2016.96) 27494293 PMC5315486

[49] Günther S, Faust K, Schumann J, Harms H, Raes J, Müller S 2016 Species-sorting and mass-transfer paradigms control managed natural metacommunities. Environ. Microbiol. 18, 4862–4877. (10.1111/1462-2920.13402) 27338005

[50] Nemergut DR et al 2016 Decreases in average bacterial community rRNA operon copy number during succession. ISME J. 10, 1147–1156. (10.1038/ismej.2015.191) 26565722 PMC5029226

[51] Jones SE, Lennon JT 2010 Dormancy contributes to the maintenance of microbial diversity. Proc. Natl Acad. Sci. USA 107, 5881–5886. (10.1073/pnas.0912765107) 20231463 PMC2851880

[52] Shoemaker WR, Lennon JT 2018 Evolution with a seed bank: the population genetic consequences of microbial dormancy. Evol. Appl. 11, 60–75. (10.1111/eva.12557) 29302272 PMC5748526

[53] Miller AD, Chesson P 2009 Coexistence in disturbance-prone communities: how a resistance-resilience trade-off generates coexistence via the storage effect. Am. Nat. 173, E30–E43. (10.1086/597669) 29553822

[54] Warner RR, Chesson PL 1985 Coexistence mediated by recruitment fluctuations—a field guide to the storage effect. Am. Nat. 125, 769–787. (10.1086/284379)

[55] Barabás G, D'Andrea R, Stump SM 2018 Chesson's coexistence theory. Ecol. Monogr. 88, 277–303. (10.1002/ecm.1302)

[56] Hubert C et al 2009 A constant flux of diverse thermophilic bacteria into the cold Arctic seabed. Science 325, 1541–1544. (10.1126/science.1174012) 19762643

[57] Portillo MC, Santana M, Gonzalez JM 2012 Presence and potential role of thermophilic bacteria in temperate terrestrial environments. Naturwissenschaften 99, 43–53. (10.1007/s00114-011-0867-z) 22159635

[58] Marchant R, Franzetti A, Pavlostathis SG, Tas DO, Erdbrugger I, Unyayar A, Mazmanci MA, Banat IM 2008 Thermophilic bacteria in cool temperate soils: are they metabolically active or continually added by global atmospheric transport? Appl. Microbiol. Biotechnol. 78, 841–852. (10.1007/s00253-008-1372-y) 18256821

[59] Blazewicz SJ, Barnard RL, Daly RA, Firestone MK 2013 Evaluating rRNA as an indicator of microbial activity in environmental communities: limitations and uses. ISME J. 7, 2061–2068. (10.1038/ismej.2013.102) 23823491 PMC3806256

[60] Dlott G, Maul JE, Buyer J, Yarwood S 2015 Microbial rRNA: RDNA gene ratios may be unexpectedly low due to extracellular DNA preservation in soils. J. Microbiol. Methods 115, 112–120. (10.1016/j.mimet.2015.05.027) 26055315

[61] Shade A, Gregory Caporaso J, Handelsman J, Knight R, Fierer N 2013 A meta-analysis of changes in bacterial and archaeal communities with time. ISME J. 7, 1493–1506. (10.1038/ismej.2013.54) 23575374 PMC3721121

