# Supplementary material for: Dormancy dynamics and dispersal contribute to soil microbiome resilience
Source: Philos Trans R Soc Lond B Biol Sci. 2020 Mar 23;375(1798):20190255. doi: 10.1098/rstb.2019.0255 (PMC7133531; doi:10.1098/rstb.2019.0255)
Supplement: Supporting results, figures and tables [file rstb20190255supp1.pdf]

Supplementary Materials for "Dormancy dynamics and dispersal contribute to soil microbiome resilience" by JW Sorensen and A Shade

Supplementary Results

Relationships between taxon activity and abundance

The conventional thought is that relative abundance is the outcome of growth and therefore an indicator of fitness, and so high relative abundance is indicative of recent or current activity in the environment. However, we detected a weak, but statistically supported, inverse (log10) relationship between OTU 16S rRNA:rRNA gene ratio and relative abundance for those taxa with an rRNA:rRNA gene ratio >1 (**Figure S2A**, Pearson's R = -.14, $p < 0.0001$). This result is in agreement with other studies that have suggested that rare taxa may have high activity levels relative to their abundance in the community [42–46]. We present it here to be transparent that there are likely additional active but rare members that contribute to stability that have not been considered in our analyses.

The inverse relationship between activity and abundance could not include taxa that had RNA but no DNA detected (aka "phantom taxa", [44]) because they have an undefined 16S rRNA:rRNA gene ratio. We make clear that, to be conservative, phantom taxa (that have RNA but no DNA detected) were not included in the analyses, and that rare taxa that had high activity ratios were not included in the description of activity response patterns among the top 50 most abundant taxa. On balance, phantom taxa contributed proportionally few rRNA reads and few unique OTUs to the dataset (**Figure S2 B and C**). However, there were a few exceptions, including five samples that had >10% rRNA reads and > 50% of richness attributed

to phantom taxa. Four of these were from the Disturbance mesocosms at week 14 (peak-thermal press), and one sample was from week 16, at the end of the press. These samples also had relatively low richness and community size (**Figure 2** and **3**). We speculate that, by reducing community size and likely also total microbial biomass, the disturbance indirectly provoked relatively higher contributions by phantom taxa and conditionally rare taxa [47].

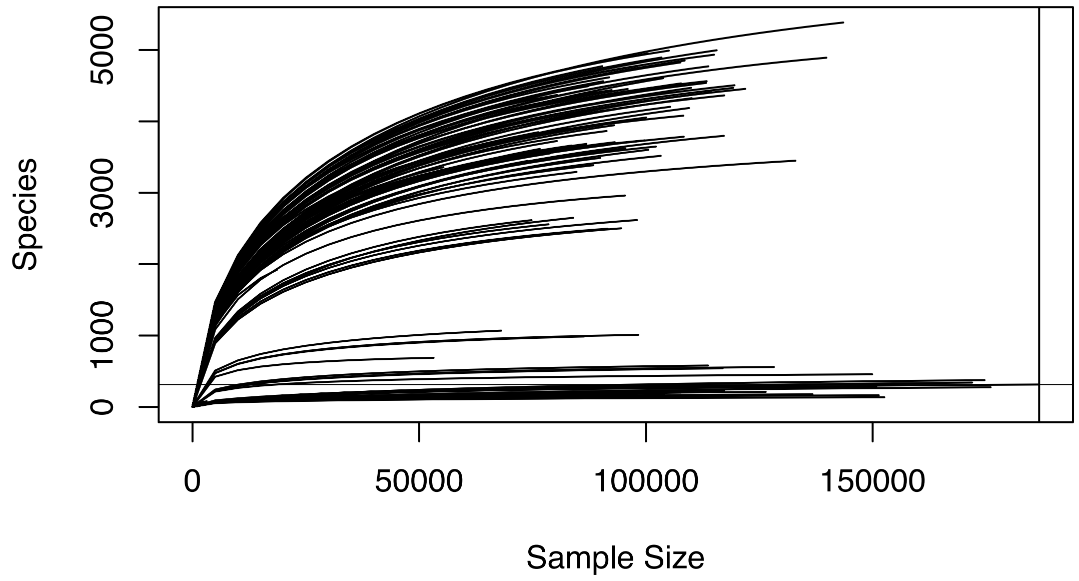

Figure S1: Rarefaction curves for soil mesocosm microbial communities.

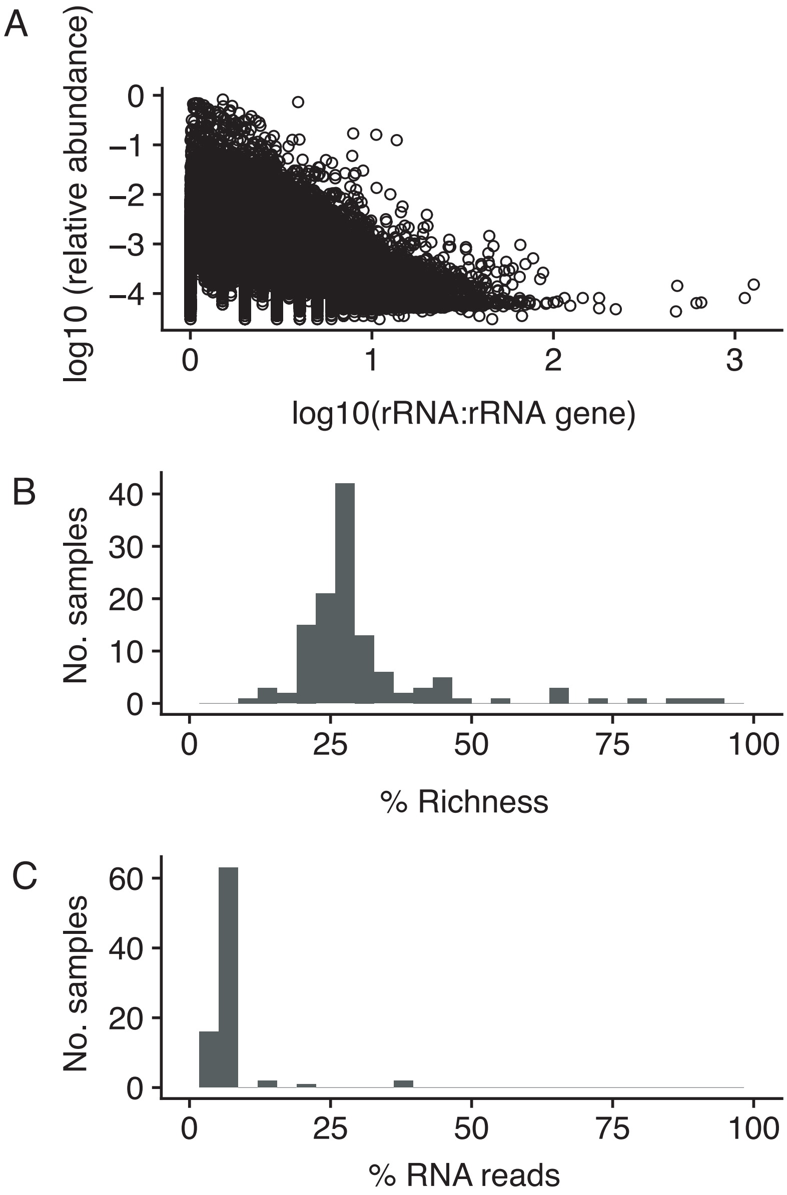

Figure S2. Taxon activity and abundance relationships. (A) Log10 relative abundance and log10 rRNA:rRNA gene ratio were inversely correlated. Each point is a different OTU detected in the dataset that had 16S rRNA:rRNA gene greater than or equal to 1. (B) Distribution of percent sample richness (No. OTUs detected, inclusive of DNA and RNA datasets) that were phantom taxa (16S rRNA detected but not 16S rRNA gene). (C) Distribution of percent RNA reads attributed to phantom taxa.

Table S1: Kruskal Wallis tests for Richness between Disturbance and Disturbance + Immigration mesocosms during the press

Week	KW rank sum statistic	p value
4	5.00	0.025
5	1.13	0.289
6	5.33	0.021
10	0.96	0.327
14	0.02	0.885
15	2.00	0.157
16	1.50	0.221

Table S2: Kruskal Wallis tests on community size between Disturbance and Disturbance + Immigration treatments during press

Week	KW rank sum statistic	p value
4	0.59	0.441
5	0.05	0.821
6	3.38	0.066
10	0.90	0.342
14	0.72	0.396
15	4.21	0.040
16	0.55	0.456

Table S3: ANOSIM tests on influence of disturbance on community structure

Week	ANOSIM R	P value
4	0.17	0.055
5	0.57	0.001
6	1.00	0.002
10	1.00	0.002
14	1.00	0.001
15	1.00	0.002
16	1.00	0.001
20	1.00	0.001
45	0.64	0.003

Table S4: ANOSIM results of community structure differences between Disturbance and Disturbance + Immigration mesocosms during the press.

Week	ANOSIM R	p value
4	0.54	0.038
5	0.15	0.222
6	-0.06	0.515
10	-0.05	0.63
14	0.07	0.449
15	0.20	0.196
16	0.04	0.359
